# Development and Validation of the First Smart TV-Based Visual Acuity Test: A Prospective Study

**DOI:** 10.3390/healthcare10112117

**Published:** 2022-10-22

**Authors:** Georgios Labiris, Konstantinos Delibasis, Eirini-Kanella Panagiotopoulou, Vassilis Pigadas, Minas Bakirtzis, Christos Panagis, Doukas Dardabounis, Panagiota Ntonti

**Affiliations:** 1 Department of Ophthalmology, University Hospital of Alexandroupolis, Dragana, 68100 Alexandroupolis, Greece; 2 Department of Computer Science and Biomedical Informatics, University of Thessaly, 35100 Lamia, Greece

**Keywords:** visual acuity test, smart TV, smartphone, home, validation, telemedicine

## Abstract

(1) Background: While smartphones are among the primary devices used in telemedical applications, smart TV healthcare apps are not prevalent despite smart TVs’ penetrance in home settings. The present study’s objective was to develop and validate the first smart TV-based visual acuity (VA) test (Democritus Digital Visual Acuity Test (DDiVAT)) that allows a reliable VA self-assessment. (2) Methods: This is a prospective validation study. DDiVAT introduces several advanced features for reliable VA self-testing; among them: automatic calibration, voice recognition, voice guidance, automatic calculation of VA indexes, and a smart TV-based messaging system. Normal and low vision participants were included in the validation. DDiVAT VA results (VA_DDiVAT_) were compared against the ones from: (a) the gold-standard conventional ETDRS (VA_ETDRS_), and, (b) an independent ophthalmologist who monitored the self-examination testing (VA_RES_). Comparisons were performed by noninferiority test (set at 2.5-letters) and intraclass correlation coefficients (ICCs). DDiVAT’s test-retest reliability was assessed within a 15-day time-window. (3) Results: A total of 300 participants (185 and 115 with normal and low vision, respectively) responded to ETDRS and DDiVAT. Mean difference in letters was −0.05 for VA_ETDRS_–VA_RES_, 0.62 for VA_RES_–VA_DDiVAT_, and 0.67 for VA_ETDRS_–VA_DDiVAT_, significantly lower than the 2.5 letter noninferiority margin. ICCs indicated an excellent level of agreement, collectively and for each group (0.922-0.996). All displayed letters in DDiVAT presented a similar difficulty. The overall accuracy of the voice recognition service was 96.01%. ICC for VA_DDiVAT_ test-retest was 0.957. (4) Conclusions: The proposed DDiVAT presented non-significant VA differences with the ETDRS, suggesting that it can be used for accurate VA self-assessment in telemedical settings, both for normal and low-vision patients.

## 1. Introduction

It is a truism that the National Healthcare Systems (NHS) are under pressure in order to address the constantly increasing ophthalmological needs of their beneficiaries. Modern lifestyle and increased life-expectancy result in an exponential increase in the overall costs of ophthalmological care. Among the primary ocular diseases that escalate care provision costs are age-related macular degeneration (ARMD) and diabetic retinopathy, since their increasing prevalence results in a growing number of patients with irreversible visual acuity (VA) damage [1,2]. Since VA reduction deteriorates the overall visual capacity, it exerts a devastating impact on the productivity and the quality of life [3,4]. Efficient management of sight-threatening diseases revealed the importance of telemedicine, which is further boosted by the technological advancements in the smart hardware and networking. Numerous telemedical services in ophthalmology have been introduced [5,6], such as the screening of diabetic retinopathy [7], ARMD [8], glaucoma [9], and amblyopia [10].

Smartphones have traditionally been used in telemedicine programs, primarily as sensor interfaces, since their high prevalence, about 80% on a worldwide scale, makes them the primary devices for continuous health-data collection [11,12]. Smart TVs are another technology with high prevalence in the general public. More than 120 million Americans owned a smart TV in 2021 [13]. Although smart TVs lack the smartphones’ mobility, their big, high-resolution screens allow for the development of diagnostic tests that cannot be performed on t small smartphone or tablet screens. Despite that fact, only a few smart TV health-related applications have been introduced, mainly lifestyle oriented [14,15].

In a hospital setting, VA is the primary clinical parameter for the screening and the diagnosis of the majority of ophthalmological diseases. Therefore, it is no surprise that VA’s importance has also been indicated in telemedical settings. Several conventional VA charts and reading tests were converted to digital applications [16,17], while others were developed solely as digital applications in order to support telemedical initiatives [18,19].

However, the telemedical screening of VA cannot be reliably performed using a smartphone or even a tablet, primarily due to the fact that the required distance between the patient and the screen is at least three meters. A full conventional VA examination requires presentation of five symbols in a row with at least logMAR 1 size. To our knowledge, none of the commercially available smartphones or tablets has a screen large enough to support logMAR 1 VA assessment.

Accordingly, it becomes obvious that smart TVs have the technical potential to support telemedical VA examination and replicate a full conventional VA testing with five symbols in a row, at distances of at least 3 m. Within this context, the primary objective of this study was to develop and validate a smart TV-based VA test (Democritus Digital Vision Acuity Test (DDiVAT)) for the reliable assessment of VA in any telemedical setting.

## 2. Materials and Methods

### 2.1. Setting

This was a prospective study that was divided into a developmental and a validation phase. The protocol adhered to the tenets of the Declaration of Helsinki, and was approved by the Institutional Review Board of Democritus University of Thrace. Written informed consent was provided by all participants. Official registration number of the study is NCT04739137.

### 2.2. Development of the Democritus Digital Visual Acuity Test-DDiVAT

DDiVAT’s study objectives required that a series of fundamental prerequisites had to be addressed: (a) In addition to assisted VA testing (i.e., by a caregiver in a remote facility), DDiVAT had to support VA self-examination; (b) In addition to normal vision patients, even low-vision ones had to be able to perform the VA self-examination; (c) No specialized hardware (other than a smart TV and a smartphone with internet connection) should be used; (d) The overall DDiVAT service should be cloud-based.

#### 2.2.1. DDiVAT System Architecture

To address the aforementioned prerequisites, DDiVAT was built as a System-as-a-Service (SAS) with the following primary components: (a) a DDIVAT administration site; (b) a smart TV application (TV-app); and (c) a smartphone app (SP-app). DDiVAT’s VA self-examination mode required several advanced services; among them: (a) voice guidance; (b) voice recognition; and (c) automatic calculation of VA scores. In detail, DDiVAT SAS consisted of:

- An Application Programming Interface (API), accessible by the cloud infrastructure. The representational state transfer—REST API—was selected. The API provides the implemented functionality to all communication between the components (TV-app, SP-app, and the administration site). It manages all related information and it provides the smart feature of voice recognition. MongoDB Atlas^TM^ was selected as the appropriate non-SQL database (SQL: Structured Query Language);
- A smart TV application (TV-app) developed in Kotlin and built for Android that offered all the functionality, except for the voice recognition feature;
- A smartphone application (SP-app) developed in Kotlin and built for Android that performed: (a) application control and navigation; (b) voice guidance; (c) communication with the voice recognition cloud service; and (d) pairing with the TV-app;
- An administrator’s site, built using framework Angular 9, available over any internet browser for every smart device such as smartphone, tablet, or PC that provided all the functionality.

**Figure 1:**
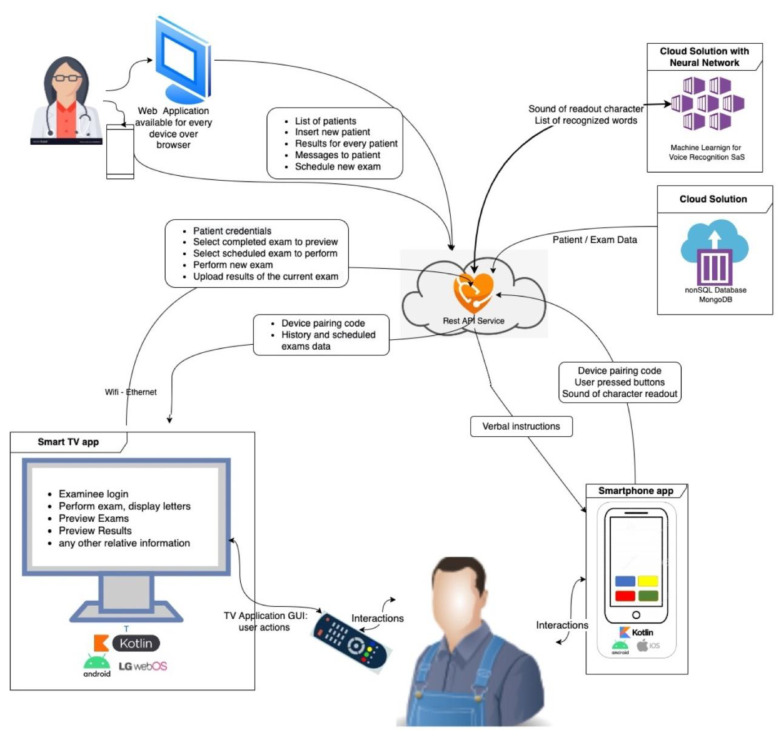
DDiVAT System-as-a-Service (SAS) explained.

**Figure 2:**
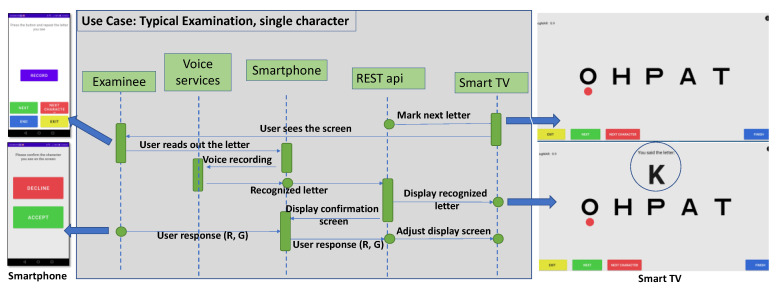
DDiVAT’s unified modeling language (UML) diagram for the voice recognition service.

Internet connections between the system’s components were implemented using websockets. DDIVAT’s SAS is presented in Figure 1. The unified modeling language (UML) diagram for the voice recognition is shown in Figure 2, along with the relevant smartphone and smart TV screens.

#### 2.2.2. Examination Modes of DDiVAT

Conventional VA tests contain a set of characters, symbols, or phrases of progressively smaller size, that are read by the patient from a predefined distance. Modern tests such as the MNREAD [20], the DDART [17,18], and the ETDRS [21] use a logarithmic scale to progressively reduce the size of the reading text by a factor of 10^−0.1^, between two consecutive sizes. The condition that must be met for VA of Snellen fraction 20/20, or 85 letters, or logMAR = 0, or visual acuity score-VAS = 100, is defined as reading text with a size such that the apparent angle of each character is δφ = 5 min of arc. The height H of a character at any logMAR, when viewed from distance D, is given by the Formula (1):

H = D tanδφ 10^logMAR^ (1)

**Figure 3:**
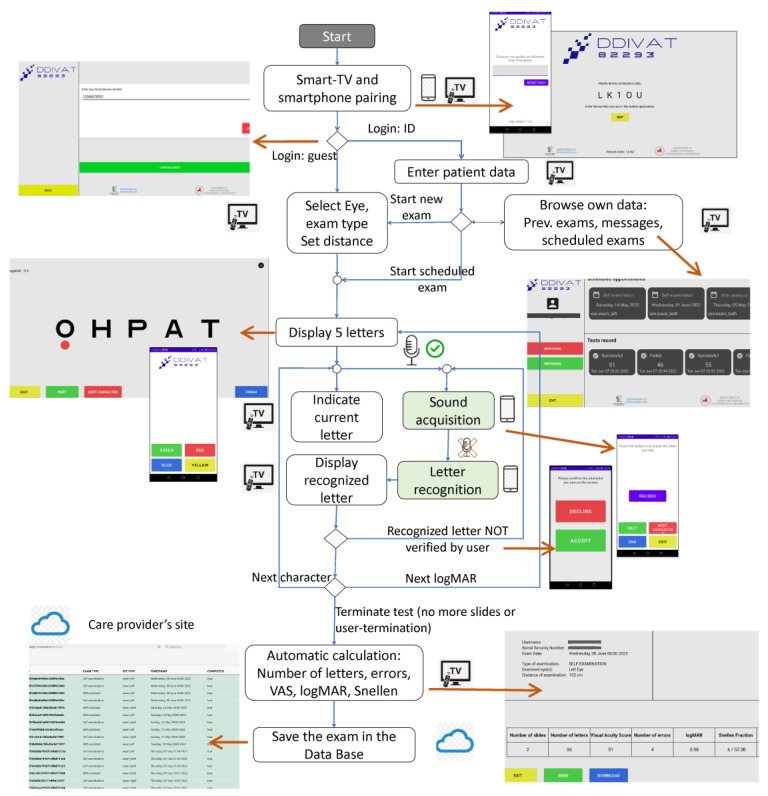
DDiVAT Control Flow chart.

DDIVAT introduced two modes for remote VA examination: (a) the operator-assisted mode (OAM) and (b) the self-examination mode (SEM). OAM requires the installation of the DDiVAT TV app (TV-app) in a smart TV, while SEM requires the installation of the TV app on a smart TV and the installation of the DDiVAT smartphone app (SP-app) on a smartphone. The TV-app and the SP-app were connected via internet to a dedicated DDiVAT server. The server established a connection between the two smart devices, managed the patients, stored VA measurements in a database, hosted a web interface for the care provider to administer the overall DDiVAT service, and enabled a chat-mode communication between the patient and the care provider through the TV-app. DDiVAT’s control flow is schematically demonstrated in Figure 3.

**Figure 4:**
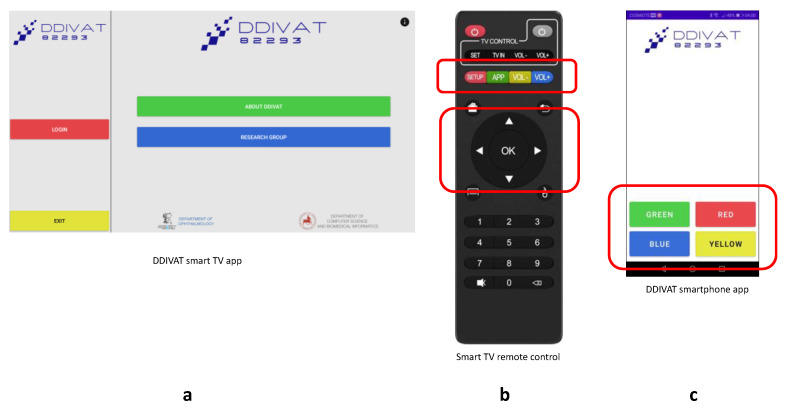
DDiVAT color-based navigation: (**a**) DDiVAT smart TV-app; (**b**) Smart TV remote control; (**c**) DDiVAT SP-app.

In OAM, a caregiver (operator) navigates DDiVAT via the TV remote control and manually inputs the examinee’s reading errors in the TV-app. In fact, OAM simulates a conventional VA examination in a remote setting. In SEM, all advanced DDiVAT features are enabled and both TV-app and SP-app work synergistically: the patient is operating DDiVAT by the smartphone, responding to verbal instructions from the SP-app, while reading errors are identified automatically by DDiVAT’s voice recognition service. To ensure that even low-vision patients with VA logMAR 1 will be able to use DDiVAT, a simple, color-based interface was designed. All user actions are performed using the four colored buttons of the smart TV remote control, or alternatively the four virtual color buttons in the smartphone screen that correspond to the same colored virtual buttons in the TV screen, as shown in Figure 4.

**Figure 5:**
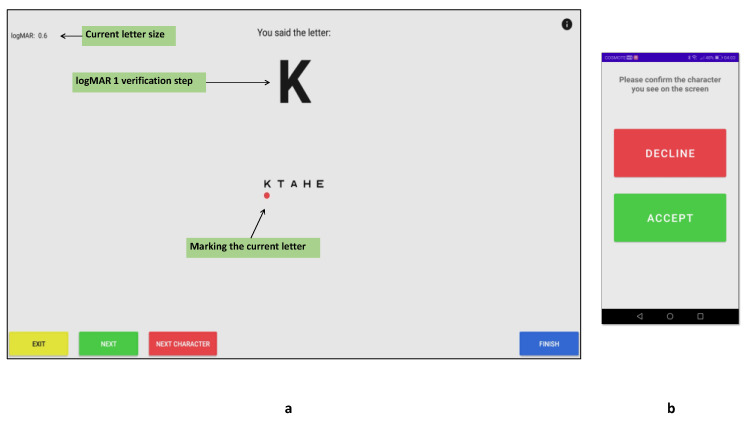
logMAR 1 verification step: (**a**) DDiVAT’s TV-app verification screen; (**b**) DDiVAT’s SP-app verification screen.

Although DDiVAT uses a high-end voice recognition service in order to identify the patient’s responses, an additional verification step has been implemented, which ensures that potential failures of the service will not compromise DDiVAT’s accuracy. The verification step suggests that each patient’s response to a letter that is identified by DDiVAT, is displayed in the TV-screen in a logMAR 1.0 size (logMAR 1 verification). Then, the examinee confirms whether the displayed letter is the letter that he/she had actually said, by pressing the corresponding button in the SP-app (Figure 5).

#### 2.2.3. Automatic Text Size Calibration of DDiVAT

In conventional clinical settings, VA assessment is usually performed at distances between 3 and 4 m (depending on the size of the examination room). According to Equation (1), a typical distance D = 300 cm requires height H of a character corresponding to logMAR = 0 to be equal to 4.36 mm. DDiVAT allows a variable, user-defined examination distance with default largest character size of logMAR = 1.0.

The TV-app automatically acquires the physical size and pixel resolution of the smart TV screen, calculates the size of the displayed letters according to the examination distance, and terminates the examination when no smaller logMAR can be displayed (each character requires at least a matrix of 5 × 5 pixels to be correctly displayed). DDiVAT allows testing up to logMAR = −0.3, provided that the TV screen has sufficient resolution.

### 2.3. Validation of DDiVAT

#### 2.3.1. Participants

Participants were enrolled from the outpatient service of the Department of Ophthalmology in a consecutive-if-eligible basis. The eligibility criteria were the following:

(1) Age between 18 and 75 years;
(2) Best spectacle-corrected distance VA (BSCDVA) ≤ 1.0 logMAR (≥35 letters);
(3) Spherical equivalent (SE) between −8.00 D and +6.00 D.

The exclusion criteria were the following:

(1) Diagnosis of neurological, mental and/or psychiatric disease, irrespective of medication for these diseases;
(2) Inability to understand study objectives;
(3) Recent eye surgery less than one month ago.

Patients with BSCDVA < 0.6 logMAR (>55 letters) populated the Normal Vision Group (NVG), while the rest of the patients populated the Low Vision Group (LVG). DDiVAT’s test-retest reliability for all participants was completed within a 15-day window.

#### 2.3.2. Examination Process—Data Collection

DDiVAT’s validation was performed using a 4K (3840 × 2160) 55-inch Android smart TV, and a 6.7-inch Android smartphone. The brightness of the TV and smartphone screen was kept constant in all tests. All measurements were made under the same conditions for all participants. BSCDVA was evaluated in one randomly selected eye for each study participant with the conventional ETDRS test [21] at a 3 m distance. This variable was named VA_ETDRS_ and was calculated in logMAR and letters. Subsequently, the participant underwent a 30-min training course on the objectives and the operation of DDiVAT and was asked to perform a self-examination (SEM) in the presence of an independent researcher, who was not allowed to interact with him/her. The researcher during SEM was writing down: (a) the letters actually displayed on the TV-app for each logMAR (Letters_TV_); (b) the letters identified by the participant (Letters_Par_); and (c) the letters recognized by the voice recognition service (Letters_DDiVAT_). At the end of the examination, DDiVAT automatically calculated BSCDVA in logMAR and letters and this variable was named VA_DDiVAT_. The researcher also calculated the score of BSCVA, which was named VA_RES_, based on the letters that each participant identified during the examination process (Letters_Par_). Summarizing, for each participant the following clinical parameters were calculated:

(1) Monocular BSCDVA measured with the conventional ETDRS (VA_ETDRS_);
(2) Monocular BSCDVA measured by the researcher through the DDiVAT examination (VA_RES_);
(3) Monocular BSCDVA automatically calculated by the DDiVAT application (VA_DDiVAT_).

Following SEM testing, each participant responded to a structured questionnaire that pertained to his/her views on the DDiVAT test, and familiarization with smart technology (Appendix A).

The main measured quantities are summarized in Table 1.

#### 2.3.3. Statistical Analysis

According to an a priori power analysis, for an effect size of 0.53 of the BSCDVA, 298 participants would be required for the study to have a power of 0.8 at the significance level of 0.05. The Shapiro–Wilk test assessed the deviation in the parameter values from the normal distribution. For the normally distributed data, the mean ± standard deviation (SD) was used, while for the non-normally distributed data, the median and the interquartile range (IQR) [25%, 75%] were used. All statistical analyses were performed with the MedCalc version 20.0.0 (Med-Calc Software, Mariakerke, Belgium).

A noninferiority test was performed between VA_ETDRS_, VA_RES_, and VA_DDiVAT_ with a margin of 2.5 letters according to former reports [22]. The level of agreement among the three VA methods was evaluated with the intraclass correlation coefficients (ICCs) and using Bland–Altman plots. Test-retest reliability of the VA_DDiVAT_ was evaluated by ICCs.

To gain further insight into the difficulty that each letter presented to the participants and the accuracy of the voice recognition service, the following confusion matrices were constructed: (a) the letters actually presented in the TV-app (Letters_TV_) versus the letters read by the participants (Letters_Par_); and (b) the letters read by the participants (Letters_Par_) versus the letters identified by the voice recognition service (Letters_DDiVAT_).

## 3. Results

From 378 enrolled participants, 79.3% fulfilled the study mandates (300 participants, 144 men, 156 women). A total of 185 populated the NVG and the remaining 115 participants the LVG, with a median BSCDVA of 0.22 logMAR and 0.78 logMAR, respectively. Median age was 69 years with non-significant differences between the NVG and LVG (*p* = 0.809). A total of 86 study participants (28.5%) had no eye pathology, 70 (23.3%) had exudative age-related macular degeneration (AMD), 18 (6.1%) non-exudative AMD, 76 (25.3%) had diabetic macular edema, 8 (2.67%) had branch retinal vein occlusion, 2 (0.67%) had retinal detachment, 24 (8%) had cataract, 4 (1.32%) had Irvine–Gass syndrome, 2 (0.67%) had macular hole, 2 (0.67%) had corneal transplantation, and 5 (16.7%) had glaucoma. A total of 51% of the NVG and 33% of the LVG participants owned a smartphone, while 42% (NVG) and 32% (LVG) had a smart TV in their home setting. Demographic characteristics and clinical parameters of the two groups are shown in Table 2.

**Figure 6:**
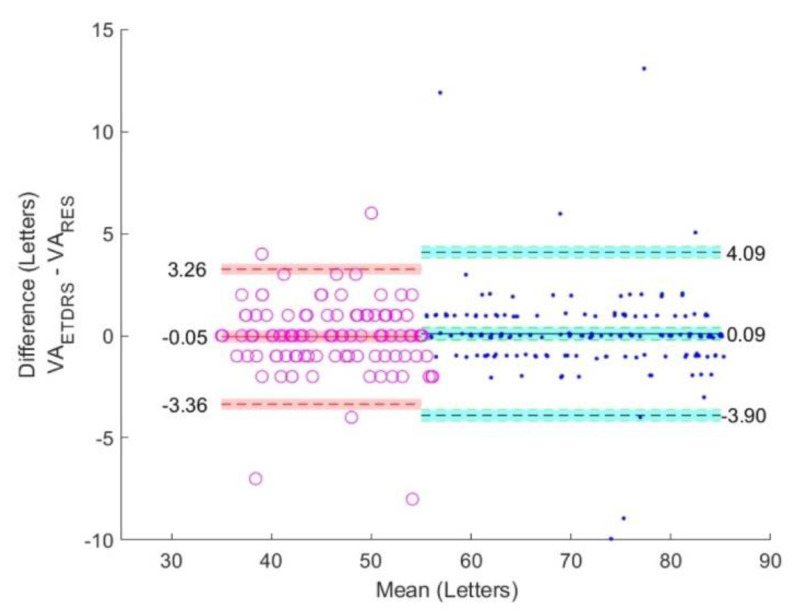
Bland-Altman plots comparing VA_ETDRS_ and VA_RES_ in NVG (blue) and LVG (red).

**Figure 7:**
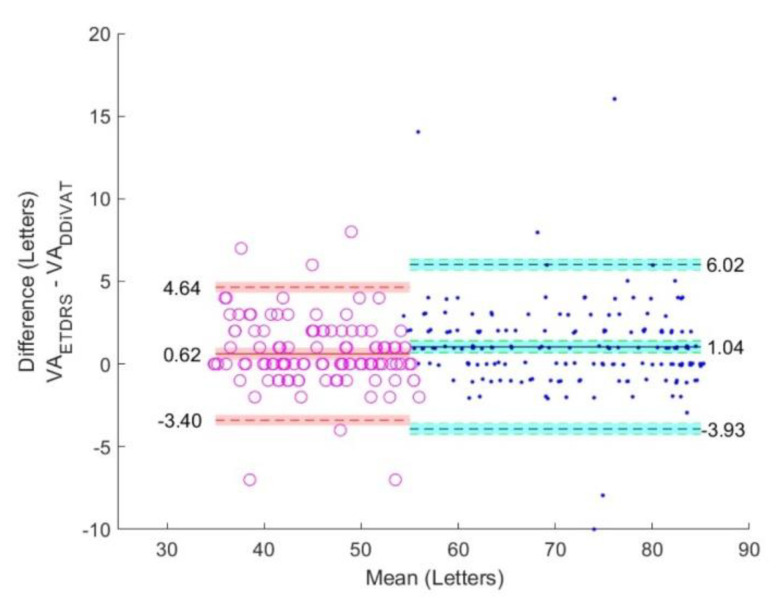
Bland-Altman plots comparing VA_ETDRS_ and VA_DDiVAT_ in NVG (blue) and LVG (red).

**Figure 8:**
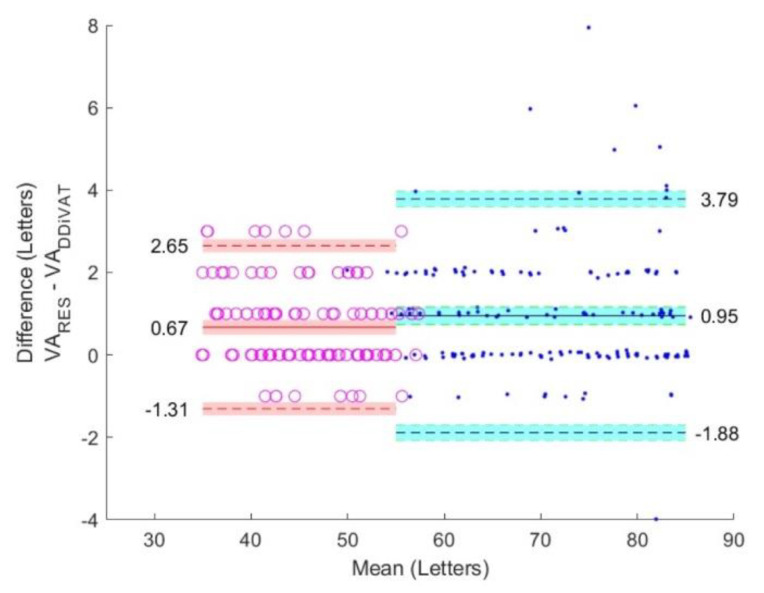
Bland-Altman plots comparing VA_RES_ and VA_DDiVAT_ in NVG (blue) and LVG (red).

The VA_ETDRS_, VA_RES_, and VA_DDiVAT_ are presented in Table 3 for all participants as well as for NVG and LVG. Figure 6, Figure 7 and Figure 8 show Bland–Altman plots evaluating differences between VA_ETDRS_ and VARES, between VA_RES_ and VA_DDiVAT_, and between VA_ETDRS_ and VA_DDiVAT_, (in letters) for both NVG and LVG.

**Figure 9:**
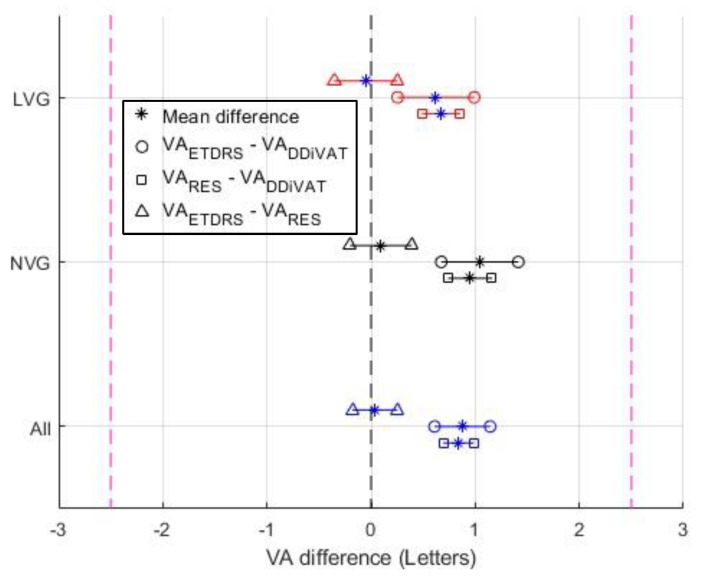
Noninferiority analysis (95% CI and mean difference value as “*”, using a 2.5-letter margin), of VA_ETDRS_ vs. VA_RES_ (denoted as “∆”), VA_ETDRS_ vs. VA_DDiVAT_ (denoted as “o”), and VA_RES_ vs. VA_DDiVAT_ (denoted as “□”) for NVG (black), LVG (red), and all patients (blue).

Mean differences in letters (VA_ETDRS_–VA_RES_), (VA_RES_–VA_DDiVAT_), and (VA_ETDRS_–VA_DDiVAT_), as well as the corresponding 95% confidence interval (CI) are presented in Figure 9. The noninferiority margin was set at 2.5 letters (equivalent to 0.05 logMAR). It becomes obvious that the CI for the difference between the VA_ETDRS_ and VA_RES_ is an almost symmetrical round 0-letter. On the other hand, the CI between VA_ETDRS_ –VA_DDiVAT_ and VA_RES_–VA_DDiVAT_ lies on the right side of the 0-letter line. This is expected since the accuracy of the voice recognition service is 96.01%, which means that: (a) a small number of letters correctly identified by the examinee will be wrongly recognized by the SP-app’s voice recognition service and accounted as an error (false negative); or (b) in extreme cases, the examinee wrongly identifies the displayed letter but the voice recognition service of the SP-app recognizes the patient response as correct (false positive). However, the latter scenario is highly improbable (probability of wrong character recognition × probability of wrongly recognizing the displayed letter = 0.039 × 1/24). Therefore, it is expected that the VA_DDiVAT_ will be consistently worse than both VA_ETDRS_ and VA_RES_, but in all cases within the 2.5-letter noninferiority margin.

ICCs and LoAs for BSCDVA are presented in Table 4. For all comparisons (VA_ETDRS_ vs VA_RES_, VA_RES_ vs VA_DDiVAT_, VA_ETDRS_ vs VA_DDiVAT_), ICCs indicated excellent level of agreement for both groups (ICCs: NVG: from 0.970 to 0.991; LVG: from 0.922 to 0.968), and for all participants (from 0.988 to 0.996).

**Figure 10:**
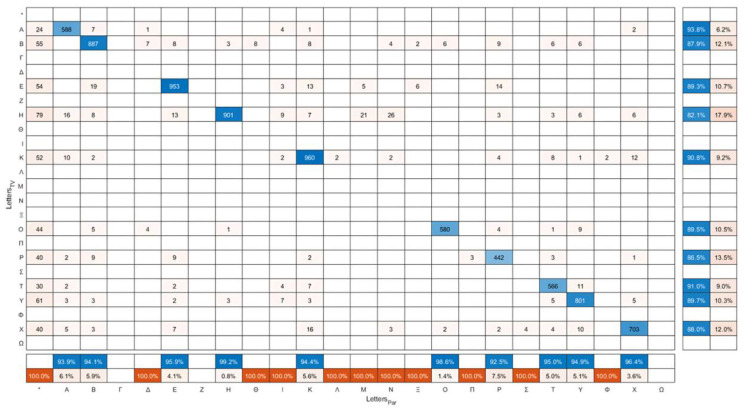
Confusion matrix of the letters displayed in DDiVAT’s TV-app (Letters_TV_) versus the letters read by the participants (Letters_Par_).

The confusion matrix of the letters displayed in the smart TV (Letters_TV_) versus the letters that were read by the participants (Letters_Par_) is presented in Figure 10. The sum of each row is the actual number of appearances of each letter in the TV-app, whereas the sum of each column is the number of times that each letter was said by the examinees. The numbers in the main diagonal correspond to the letters that were correctly identified by the examinees. It has to be mentioned that, although DDiVAT displays only 10 Latin characters (A, B, E, H, K, O, P, T, Y, X), the confusion matrix contains almost all the characters of the alphabet, since the examinees occasionally incorrectly read non-displayed characters. This could be verified by the fact that only the rows of the aforementioned Latin characters have a non-zero sum. The star symbol “*” indicates cases in which the examinee declared that he/she was unable to read the displayed letter. The true positive rate (TPR) (or recall, or sensitivity) and false negative rate (FNR) in examinee-reading for each letter is summarized in the additional column on the rightmost of the confusion matrix. The row at the bottom of the confusion matrix shows the positive predicted value (PPV) or precision, and the false discovery rate (FDR) of patient-reading for each letter.

**Figure 11:**
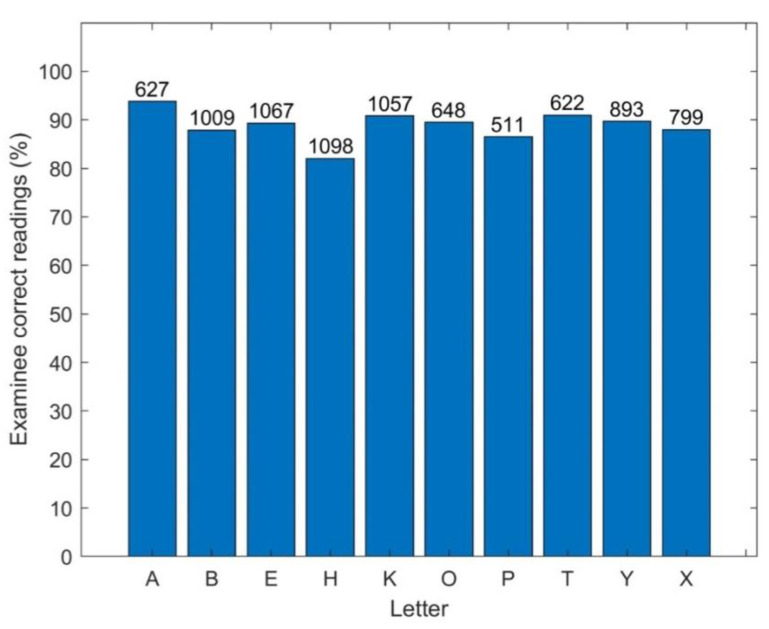
Percentage of correct identification for each letter by the examinees.

All displayed letters by the TV-app presented a similar difficulty to the examinees, as demonstrated in Figure 11, which shows the percentages of correct read out of each letter.

**Figure 12:**
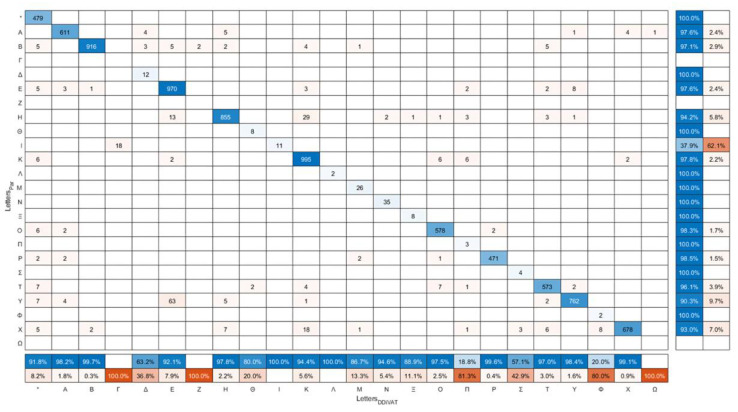
Confusion matrix of the letters read by participants (Letters_Par_) versus the letters recognized by the smartphone (Letters_DDiVAT_).

The confusion matrix of the letters read by both NVG and LVG participants (VA_RES_) versus the letters that were automatically recognized by the SP-app (VA_DDiVAT_) is presented in Figure 12. The sum of each column is the number of times the specific letter was recognized by the voice recognition service. The sum of each row is the number of times the specific letter was read by the participants, which should be equal to the sum of the corresponding column of the confusion matrix in Figure 10. The column on the right side summarizes the true positive rate (TPR) (or recall, or sensitivity) and false negative rate (FNR) in automatic letter recognition by the smartphone. The row at the bottom of the plot shows the positive predicted value (PPV) or precision, and the false discovery rate (FDR) of letter recognition.

**Figure 13:**
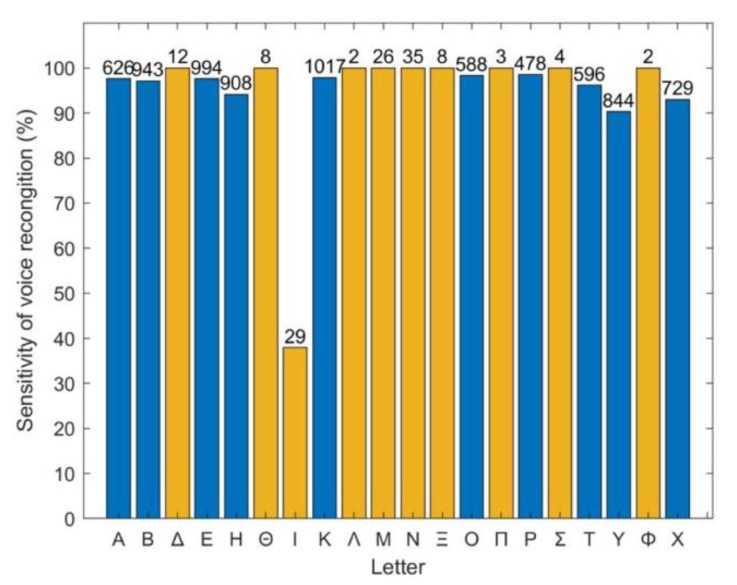
Percentage of letters correctly identified by the voice recognition service (sensitivity). The number of each letter’s appearance is shown at the top of each column. Blue bars: Latin letters used in the test and appearing in TV-app; orange bars: letters not included in the test, thus not appearing in TV-app.

The sensitivity of the voice recognition service is presented in Figure 13. The majority of the letters was identified with over 90% sensitivity, except from letter “I”; however, with no apparent impact on DDiVAT’s reliability since “I” is not among the letters included in the VA test.

Participants’ responses to the questionnaire and comparisons with their demographic profile are presented in Table 5. Younger age, male gender, smartphone and smart TV use were associated with better readiness to use a telemedical application such as the DDiVAT.

The ICC for the test-retest of the VA_DDiVAT_ was 0.957. Of the 300 participants that completed the SEM re-testing within the 15-day window, 34 had to repeat the 30-min training course to refamiliarize with the test mandates.

## 4. Discussion

It is a truism that there is a growing demand for digital healthcare services. According to conservative estimates, more than 200,000 health-related applications were available on iTunes and Google Play stores in 2018 and their number of downloads increased from 1.3 billion in 2013 to 3.7 billion in 2017 [23]. Moreover, in 2017 more than 75% of Americans stated that mobile technology was important to managing their health.

Despite the fact that smart TVs show impressive prevalence to Western societies, smart TV health-related applications primarily focus on lifestyle. Unlike smartphones, smart TVs have not been used as clinical data-collecting devices [24], although their big, high-contrast, and high-resolution screens make them ideal for displaying text and/or symbols for distance vision acuity examination. It is known that in distance VA examination, the height H of a character or a symbol at any logMAR, when viewed from distance D, is derived from Formula (1). Since a distance VA examination requires the presentation of one line of five characters or symbols, a length *L* of at least 10 characters is required (assuming square fonts). Therefore, the length *L* can be calculated by Formula (2):

L = 10H = 10∙D∙tanδφ∙10^logMAR^, (2)

Assuming a screen with an aspect ratio of 16:9, the diagonal size *d* in inches is derived in the following Formula:

(3) $$d=\frac{1}{2.54}\sqrt{L^{2}+{\left(\frac{9}{16}L\right)}^{2}}$$

which can be further simplified:

(4) $$d=\frac{L}{2.54}\frac{1}{16}\sqrt{337}$$

Applying Equation (4) in a conventional distance VA examination setting at 3 or 4 m, we can easily assess the minimal size of the screen for a full testing (Table 6).

It becomes obvious that a screen size of 20.3 inches is the absolute minimum for a full distance VA examination.

Regardless of the technical details of the healthcare apps, their reliability should be supported by evidence-based medical outcomes. However, the majority of Western countries lack regulatory oversight for the provided digital healthcare apps, while global access to them makes their regulation even more difficult. Questionable reliability of any digital healthcare app could mislead the general public and contribute to poor overall disease management. Therefore, only validated apps can ensure the accuracy of their measured outcomes [25].

To alleviate concerns on the accuracy of DDiVAT’s VA measurements, we applied a validation phase against ETDRS, the gold-standard VA test, allowing a 2.5 letter non inferiority margin [22], which is extremely strict even for repeated ETDRS testing in clinical settings. The self-assessment mode of DDiVAT that incorporates a high-end voice recognition service achieved a two letter difference, which is highly acceptable both for clinical and for research settings. The non-significant difference between DDiVAT and ETDRS measurements was achieved by: (a) the automatic identification of the smart TV’s screen characteristics for the accurate display of letters according to the examinee’s distance; and (b) the logMAR 1 voice recognition verification step that allows the examinee to repeat his/her response when needed.

This study combines smart TV and cloud infrastructure that creates new possibilities in the screening and follow-up of ophthalmological and systemic diseases that present with reduced visual acuity. VA is the fundamental clinical parameter for the screening and follow-up of ARMD and diabetic retinopathy, the leading causes for irreversible damage to the visual capacity in Western societies. Smart TVs play a crucial role in home care, since they are the most convenient gadget for entertainment, news briefing, and communication for seniors, who are the primary target population for sight-threatening diseases. With the validation of the DDiVAT medical application, smart TVs become a reliable medical data-collecting device. Thus, the importance of DDiVAT becomes self-evident [1,2].

However, seniors are not the only target population for reliable smart TV-based VA testing. Smart TVs and tablets are the first technological gadgets that preschool minors become familiar with [26]. In fact, DDiVAT’s operator-assisted mode could be used for visual acuity examination of preschool minors, with his/her guardian or teacher as the application’s operator. Therefore, it may contribute to the prevention of amblyopia and vision-related learning disabilities, by increasing the awareness of the parents, especially in vulnerable populations [10,27].

The role of reliable smart TV based VA self-testing becomes even more important in pandemics that result in major reductions in ophthalmological services with many beneficiaries omitting necessary care, because of fear of infection, inability to access, or cancelation of health services [28,29,30]. It is a common belief that in pandemics such as the COVID-19 one, large populations were subjected to unnecessary or inappropriate care, with potentially significant harm to their visual capacity.

However, DDiVAT offers more advantages than accurate VA measurements, which are listed below: (a) the OAM attempts to engage the examinee and the family with the eye disorder, which facilitates optimal disease management, especially in chronic diseases [18]; (b) DDiVAT’s innovative bidirectional communication in which the physician’s messages appear on the patient’s TV screen fosters the patient–doctor bond; and (c) the global access to DDiVAT and the automatic voice recognition service could support multilanguage screening initiatives with minimal modifications to the application.

The novelty of this study which reports on a smart TV-based distance VA examination excludes direct comparisons with former similar publications. Despite that fact, an extensive literature review was attempted. The validation of a web-based application for the assessment of distance VA and of the refractive error was recently reported [31], using a smartphone and a computer. Unfortunately, the results of the study showed lower reliability and accuracy, especially in low-vision patients. The validation of the T-Assito and the Motiva projects which use a smart TV were also recently reported. T-Assito included emergency calls and notifications related to users’ health, while the Motiva introduced a smart TV-based service for monitoring vital signs in patients with chronic diseases [19].

Certain limitations of the study should be taken into consideration prior to the interpretation of our outcomes. Although DDiVAT is linguistically adaptable to any European country, it was validated in Greek-speaking populations. SEM testing in other European languages should be treated with caution, unless a validation study confirms the accuracy of the voice recognition service in that particular language. However, DDiVAT’s OAM can be safely used in any telemedical setting, regardless of the language of the subject population. Finally, it has to be noted that any digital chart cannot assess refractive errors, outside clinical settings and without specialized healthcare personnel.

## 5. Conclusions

In conclusion, the results show that DDiVAT is smart TV application that provides reliable distance VA measurements, both in normal and low-vision patients. Our validation outcomes suggested comparable reliability with the ETDRS which is the gold-standard for distance VA examination. However, contrary to the ETDRS, DDiVAT supports self-examination, and can be used in any remote setting, provided that a smart TV and an internet connection are present. Within this context, DDiVAT introduces a new potential in teleophthalmology both for screening and follow-up initiatives.

## Figures and Tables

**Table 1:** Definition and measurement of main variables.

Variable Name	Definition	Source	Measurement Method
Letters_TV_	The letters, displayed on the TV screen	Retrieved from database, also noted by the researcher	N/A*
Letters_Par_	The letters, read by the participant from the TV screen	Researcher	N/A
Letters_DDIVAT_	The letters identified by the voice recognition service on patients’ readouts	Retrieved from database, also noted by the researcher	N/A
VA_ETDRS_	Monocular BSCD Visual acuity	Researcher	Conventional ETDRS chart
VA_RES_	BSCDVA	Researcher	Using patients’ readout of DDiVAT chart
VA_DDiVAT_	BSCDVA	DDiVAT	Automated calculation

* N/A: Not applicable

**Table 2:** Demographic Characteristics and Clinical Parameters.

Group	*n* ^3^ (%)	Age (Years) Median [IQR ^4^]	Gender M ^5^/F ^6^	VA_ETDRS_^7^ Median [IQR]	Smartphone Users Yes/No	Smart TV Users Yes/No
Total	300	69 [60, 77.25]	145/155	0.43 [0.11, 0.72] logMAR 63.5 [49, 79.5] letters	133 (44%)/167 (56%)	114 (38%)/186 (62%)
NVG ^1^	185 (61.7%)	70 [61, 77]	88/97	0.22 [0.06, 0.38] logMAR 74 [82, 66] letters	95 (51%)/90 (49%)	78 (42%)/107 (58%)
LVG ^2^	115 (38.3%)	67.5 [52, 77.5]	57/58	0.78 [0.68, 0.88] logMAR 46 [41, 51] letters	38 (33%)/77 (67%)	36 (32%)/79 (68%)

^1^ Normal Vision Group; ^2^ Low Vision Group; ^3^ Number of participants; ^4^ Interquartile Range; ^5^ Male; ^6^ Female; ^7^ Visual Acuity.

**Table 3:** Comparison of median [IQR ^1^] of BSCDVA ^2^ (in logMAR).

Participants	VA_ETDRS_^5^	VA_RES_	VA_DDiVAT_
Total	0.43 [0.12, 0.72]	0.44 [0.14, 0.73]	0.47 [0.16, 0.75]
NVG ^3^	0.22 [0.06, 0.42]	0.24 [0.06, 0.42]	0.26 [0.08, 0.44]
LVG ^4^	0.78 [0.68, 0.88]	0.78 [0.68, 0.88]	0.78 [0.70, 0.92]

^1^ Interquartile Range; ^2^ Best Spectacle-Corrected Distance Visual Acuity; ^3^ Normal Vision Group; ^4^ Low Vision Group; ^5^ Visual Acuity.

**Table 4:** Intraclass correlation coefficients for study participants.

BSCDVA ^1^		NVG ^6^	LVG ^7^	Total
VA_ETDRS_^2^ vs. VA_RES_	ICC ^3^	0.981	0.961	0.993
	95% CI ^4^	[0.975, 0.986]	[0.945, 0.973]	[0.991, 0.995]
	LoA ^5^	[−0.081, 0.075]	[−0.068, 0.062]	[−0.076,0.069]
VA_ETDRS_ vs. VA_DDiVAT_	ICC	0.970	0.922	0.988
	95% CI	[0.941, 0.982]	[0.831, 0.958]	[0.974, 0.993]
	LoA	[−0.111, 0.066]	[−0.107, 0.060]	[−0.109, 0.063]
VA_RES_ vs. VA_DDiVAT_	ICC	0.991	0.968	0.996
	95% CI	[0.943, 0.996]	[0.858, 0.987]	[0.977, 0.998]
	LoA	[−0.061, 0.023]	[−0.069, 0.028]	[−0.064, 0.025]

^1^ Best Spectacle-Corrected Distance Visual Acuity; ^2^ Visual Acuity; ^3^ Intraclass Correlation Coefficient; ^4^ Confidence Interval; ^5^ Limits of Agreement; ^6^ Normal Vision Group; ^7^ Low Vision Group. Notes: ICC: two-way mixed model with measures of absolute agreement-single rating.

**Table 5:** Responses of study participants.

Questions	Gender			AGE			^1^ BSCDVA			Smartphone User			Smart TV User		
	Male (Median [IQR ^2^])	Female (Median [IQR])	*p* Value	<69 Years (Median [IQR])	≥69 Years (Median [IQR])	*p* Value	^3^ NVG (Median [IQR])	^4^ LVG (Median [IQR])	*p* Value	User (Median [IQR])	No User (Median [IQR])	*p* Value	User (Median [IQR])	No User (Median [IQR])	*p* Value
Q1	5 [4, 5]	4 [4, 5]	0.03 *	5 [4, 5]	4 [4, 5]	<0.01 *	4 [4, 5]	4 [4, 5]	0.91	5 [4, 5]	4 [4, 5]	<0.001 *	5 [4, 5]	4 [4, 5]	<0.01 *
Q2	5 [3.75, 5]	4 [3, 5]	0.04 *	5 [4, 5]	4 [4, 5]	<0.01 *	4 [4, 5]	4 [4, 5]	0.78	5 [4, 5]	4 [3, 5]	<0.001 *	5 [3.25, 5]	4 [3, 5]	0.06
Q3	4 [3, 5]	4 [3, 5]	0.05	4 [4, 5]	4 [3, 5]	0.03 *	5 [4, 5]	5 [4, 5]	0.63	4 [3, 5]	4 [3, 5]	0.13	4 [3, 5]	4 [3, 5]	0.81
Q4	5 [4, 5]	4 [3, 5]	<0.01 *	5 [4, 5]	4 [3, 5]	<0.001 *	4 [4, 5]	4 [4, 5]	0.35	5 [4, 5]	4 [3, 5]	<0.0001 *	5 [4, 5]	4 [4, 5]	0.02 *
Q5	5 [4, 5]	4 [3, 5]	0.03 *	5 [4, 5]	4 [3, 5]	<0.01 *	4 [3, 5]	4 [4, 5]	0.57	5 [4, 5]	4 [3, 5]	<0.001 *	5 [4, 5]	4 [3.5, 5]	0.07
Q6	5 [4, 5]	4 [4, 5]	<0.01 *	5 [4, 5]	5 [4, 5]	0.01 *	5 [4, 5]	5 [4, 5]	0.49	5 [4, 5]	4 [4, 5]	<0.01 *	5 [4, 5]	4 [4, 5]	0.03 *
Q7	5 [4, 5]	5 [4, 5]	0.02 *	5 [4, 5]	5 [4, 5]	0.23	5 [4, 5]	5 [4, 5]	0.83	5 [4, 5]	5 [4, 5]	0.04 *	5 [4.25, 5]	5 [4, 5]	0.02 *
Q8	5 [4, 5]	4 [4, 5]	0.20	5 [4, 5]	4 [3, 5]	0.02 *	4 [4, 5]	4 [4, 5]	0.74	5 [4, 5]	4 [3, 5]	0.01 *	5 [4, 5]	4 [3.75, 5]	0.11
Q9	5 [4, 5]	4 [3, 5]	0.03 *	5 [4, 5]	4 [3, 5]	<0.01 *	4 [4, 5]	4 [3, 5]	0.84	5 [4, 5]	4 [3, 5]	<0.0001 *	5 [4, 5]	4 [3, 5]	<0.01 *
Q10	5 [4, 5]	4 [4, 5]	<0.01 *	5 [4, 5]	4 [4, 5]	0.04 *	5 [4, 5]	5 [4, 5]	0.76	5 [4, 5]	4 [4,5]	0.01 *	5 [4, 5]	4 [4, 5]	0.25

^1^ Best Spectacle-Corrected Distance Visual Acuity; ^2^ Interquartile Range; ^3^ Normal Vision Group; ^4^ Low Vision Group. * *p* values < 0.05.

**Table 6:** Required screen diagonal (inches).

Examination Distance	logMAR = 1.3	logMAR = 1
*D* = 300 cm	39.2	20.3
*D* = 400 cm	52.3	26.2

## Data Availability

Not applicable.

## Abbreviations

API	Application Programming Interface
ARMD	Age-Related Macular Degeneration
BSCDVA	Best Spectacle-Corrected Distance Visual Acuity
CI	Confidence Interval
COVID-19	Coronavirus Disease 19
DDART	Democritus Digital Acuity & Reading Test
DDiVAT	Democritus Digital Vision Acuity Test
ETDRS	Early Treatment of Diabetic Retinopathy Study
FDR	False Discovery Rate
FNR	False Negative Rate
ICC	Intraclass Correlation Coefficient
IQR	interquartile range
LoA	Limits of Agreement
logMAR	Logarithm of the Minimum Angle of Resolution
LVG	Low Vision Group
MNREAD	Minnesota Low Vision Reading Test
NHS	National Healthcare Systems
NVG	Normal Vision Group
OAM	Operator-Assisted Mode
PC	Personal Computer
PPV	Positive Predicted Value
REST	Representational State Transfer
SAS	System-as-a-Service
SD	Standard Deviation
SEM	Self-Examination Mode
Smart TV	Smart Television
SP-app	Smartphone app
SQL	Structured Query Language
TPR	True Positive Rate
UML	Unified Modeling Language
VA	Visual Acuity
VAS	Visual Acuity Score

## Appendix A

Questionnaire
